# Music Perception Influences Language Acquisition: Melodic and Rhythmic-Melodic Perception in Children with Specific Language Impairment

**DOI:** 10.1155/2015/606470

**Published:** 2015-10-05

**Authors:** Stephan Sallat, Sebastian Jentschke

**Affiliations:** ^1^Justus-Liebig-Universität, Gießen, Germany; ^2^Speech and Language Pedagogy and Pathology, Department of Special and Inclusive Education, Faculty of Education, University of Erfurt, Nordhäuser Strasse 63, 99089 Erfurt, Germany; ^3^Freie Universität Berlin, Cluster “Languages of Emotion”, Habelschwerdter Allee 45, 14195 Berlin, Germany

## Abstract

Language and music share many properties, with a particularly strong overlap for prosody. Prosodic cues are generally regarded as crucial for language acquisition. Previous research has indicated that children with SLI fail to make use of these cues. As processing of prosodic information involves similar skills to those required in music perception, we compared music perception skills (melodic and rhythmic-melodic perception and melody recognition) in a group of children with SLI (*N* = 29, five-year-olds) to two groups of controls, either of comparable age (*N* = 39, five-year-olds) or of age closer to the children with SLI in their language skills and about one year younger (*N* = 13, four-year-olds). Children with SLI performed in most tasks below their age level, closer matching the performance level of younger controls with similar language skills. These data strengthen the view of a strong relation between language acquisition and music processing. This might open a perspective for the possible use of musical material in early diagnosis of SLI and of music in SLI therapy.

## 1. Introduction

Despite the complexity of language, most children successfully acquire the capacity to perceive and comprehend it, as well as produce spoken utterances. However, a considerable number of two-year-old children are delayed in crucial aspects of language acquisition such as vocabulary, grammar, or correct articulation. Whereas half of them make up this delay by about three years of age, approximately seven percent of an age cohort continue to have difficulties to acquire their native language without any obvious primary dysfunctions (such as mental, neurological, sensorial, oral-motor disorders; cf. [1]). These children, diagnosed with Specific Language Impairment (SLI), show deficits mainly in grammar processing (e.g., morphosyntax), phonology, and word learning. Because of their impaired language, they form a high-risk group for problems in school, as well as in other cognitive or social-emotional areas [2–5].

There is discussion regarding risk factors and possible causes for SLI in the literature [6–8]; for an overview, see [3]. However, none of these accounts cover all aspects of impaired linguistic and nonlinguistic functions in people with SLI. An interdisciplinary perspective might help to gain further insight into the aetiology of SLI. Such an approach, a comparison of speech and music perception in children with SLI and those with typical language development, is presented in this paper.

Several theoretical accounts proposed that, particularly during early language acquisition, language is rather perceived as music. For example, Koelsch ([9], p. 16) hypothesized that “the human brain, particularly at an early age, does not treat language and music as strictly separate domains, but rather treats language as a special case of music.” Brandt et al. ([10], p. 5) denoted “that music has a privileged status that enables us to acquire not only the musical conventions of our native culture, but also enables us to learn our native language.” In addition, music and language share a number of similarities (for overviews, see [9–12]). Both are based on acoustic information, involving a limited number of categorical elements or classes (phonemes and tones) that are organized in structured sequences according to specific regularities. These regularities are acquired using similar learning mechanisms [13]. There are indicators for common evolutionary origins [14, 15]. Electrophysiological evidence suggests shared cognitive resources and similar underlying neural substrates for processing semantics [16], syntax [17, 18], and prosody [19, 20].

Prosody is presumably the area with the strongest overlap: Prosodic or suprasegmental features can be regarded as “musical” aspects of the speech signal. Prosody has many functions, such as indicating the emotional state of the speaker, indicating syntactic structure, or indicating cues for turn-taking in conversations. Components of prosody (such as speech rhythm, speech melody, contour, timbre, pauses, and stress) emerge from a combination of acoustic features such as pitch/frequency, loudness/intensity, duration, and timbre [21]. During language acquisition, these prosodic components help the infant to detect word and phrase boundaries. This enables them to acquire regularities about the arrangement of linguistic patterns like phonemes, words, and phrases (for reviews, see [22, 23]). Unlike typically developing children, children with SLI appear not to profit from exaggerated prosody (contour, stress, and pauses) while learning words and grammatical rules [24, 25]. Related to music perception and prosody is a group of theories which propose deficient auditory processing (especially fine-grained temporal processing of auditory information) to account for the problems of children with SLI [26–29]. However, other authors failed to observe such problems [30–32]. More recently, the focus shifted towards auditory features which are crucial for the processing prosodic cues: Corriveau et al. [33] proposed that the accurateness of prosodic processing in SLI is impaired because the children fail to use auditory cues required for the perception of rhythm and stress, which has consequences for their language development. Przybylski et al. [34] provided evidence that children with SLI, and dyslexia, as well as controls with typical language development show better performance in grammaticality judgements after rhythmically regular than after irregular prime sequences. Although the performance level of the clinical groups was generally lower than that of the controls, they still profit from the metrical structure of the regular prime. Recently, Cumming et al. [35] proposed in their “prosodic phrasing” hypothesis that problems with processing certain acoustic properties (particularly amplitude rise time and duration), relevant for both language and music perception, may be responsible for morphosyntactical problems in children with SLI. They also observed that children with SLI were less sensitive to all auditory measures explored in the study.

In order to perceive music, children have to acquire implicit knowledge about musical structure. Two key aspects are pitch and temporal organization (cf. [18, 19]). In addition, children acquire explicit knowledge, for example, the tune of a particular song and its lyrics. A number of studies explored music perception skills in children with SLI: Jentschke et al. [36] demonstrated that five-year-old children with SLI are impaired in certain aspects of music perception, namely, in that they lack a neurophysiological marker of music-syntactic processing whereas this marker can be observed in children with typical language development. Other studies investigated music production skills in SLI. Corriveau and Goswami [37] showed that ten-year-old children with SLI were impaired in rhythmic tapping to an externally paced source but less to an internally generated rhythm and that the severity of impairment was linked to language and literacy outcomes. Recently, Clément et al. [38] investigated singing in eleven-year-old children with SLI. Compared to children of the same age but with typical language development, children with SLI were poorer in reproducing similar tunes (pitch matching) and in reproducing familiar and unfamiliar melodies. Based upon their findings, a general auditory-motor dysfunction in the children with SLI was proposed.

The present study aimed to add knowledge of whether children with SLI differ from typically developing children with regard to music perception: Skills in pitch organization were explored in a melodic perception task, those of temporal organization were explored in a rhythmic-melodic perception task, and the recognition of musical sequences stored in long-term memory was explored in a melody recognition task. Familiarity and features like tempo, sound, and pitch of these melodies were manipulated. We expected that children with SLI would, similar to their performance in the language domain, lag behind their age-matched peers and perform rather like younger children with typical language development. Such pattern would indicate a relation of music perception and linguistic skills in children with SLI and provide further evidence for a privileged status of music perception skills during language acquisition.

## 2. Methods

### 2.1. Participants

Children with specific language impairment (SLI) were compared to a control group with typical language development of the same age (controls with comparable age (CA)) and a group of younger children, whose linguistic abilities were comparable to the SLI group (younger controls with comparable language skill (CL)). Written informed consent was obtained from the parents of all participating children. A questionnaire, providing information about language development, social background, and musical environment of their children, revealed that groups did not differ in variables reflecting social (e.g., number of books or CDs in the household) or musical (e.g., family members playing an instrument; amount of singing with children) background. To determine the socioeconomic background of the children's families, the occupation of the parents was classified in terms of the “International Standard Classification of Occupation 1988” [39]. This classification was then transformed into “International Socio-Economic Index of Occupational Status” values (ISEI [40]) to provide a status measure for each occupation. There was neither a group difference for duration of school education (the vast majority of all parents attended school for 10 years) nor a group difference for professional qualification. However, whereas fathers' occupation was similar among groups (*p* > 0.50), the status value of mothers' occupation was lower in the children with SLI compared to both control groups (*p* < 0.01). An overview is given in Table 1.

Children with SLI were recruited from a kindergarten for special education (for children with language and speech disorders); children with typical language development (CA, CL) came from four public kindergartens. All kindergartens were located in Leipzig, Germany. Children (of any group) were excluded from the study when [a] their parents or teachers reported defective hearing or a history of hearing disease, [b] they did not grow up in monolingual families, [c] they had any other speech or language disorder such as oral fluency disorder (e.g., stuttering), [d] they had any other condition (mutism, autism, etc.), or [e] they had a nonverbal IQ below the low average range (i.e., less than 80 IQ points). The data of 29 children with SLI (4;8 to 5;11 years old, *M* = 5;4 years), 39 CA children (4;9 to 5;11 years old, *M* = 5;3 years), and 13 CL children (4;0 to 4;7 years old, *M* = 4;3 years) were evaluated. Like many previous studies (see, e.g., [3]), a higher incidence of SLI was observed in boys (65.5%). We tried to match this proportion in the control groups (CA: 56.4%; CL: 61.5%). The results for the language screening within the SLI group corresponded to the norms of an age group between 3;0 and 3;6 years. However, younger controls with comparable language skills (CL) were only about one year younger than the SLI children, because for younger children the music perception tasks would have been too difficult. The performance of these controls (i.e., their raw scores) in all subtests of the language screening was significantly above the SLI children (*p* < 0.030), indicating a higher semantic-lexical and morphosyntactic knowledge. Whereas the linguistic performance within the CL group lies between the SLI and the CA group, the SLI and the CL group had similar performance levels at phonological analysis (assessed by phoneme discrimination skills).

### 2.2. Stimuli and Paradigm

All measurements were acquired in either four (CA group) or five (SLI and CL group) experimental sessions of approximately 20- to 25-minute length. One session contained the language screening; in another nonverbal intelligence was tested. Linguistic skills were investigated with a standardized German language screening for three- to five-year-old children (SETK 3–5; [41]). It contained three parts: language comprehension (manipulation tasks with different objects), speech production (applying morphological rules to words and nonwords), and working memory (repetition of nonwords and sentences). In addition, phonemic discrimination (e.g., “Kanne” [pitcher] versus “Tanne” [fir]) was tested. Nonverbal intelligence was assessed using the Kaufman Assessment Battery for Children (K-ABC [42]).

In the remaining sessions, musical skills were evaluated, using tasks developed by the authors of this study. The tasks explored melodic perception, rhythmic-melodic perception, and melody recognition. Stimuli were created as MIDI files containing the beginning phrases of nursery rhymes (proposed by the kindergarten teachers and well known to all participating children). The MIDI files were exported into WAV files with a piano sound (using Steinberg Cubase SX and The Grand, Steinberg Media Technology, Hamburg, Germany). These were presented on a laptop, using Presentation 0.76 (Neurobehavioral Systems, Inc., Albany, CA), which was also used to record the answers. The suitability and age-appropriateness of stimuli and procedure were checked in a pilot test with 10 typically developing children. The pilot test also served to determine an optimal speed to present the stimuli sequences (135 beats per minute for melodic perception; 120 beats per minute for rhythmic-melodic perception).

Children sat in front of a laptop with two external speakers and listened to the stimuli. Before the experiment, children listened to the beginnings of the nursery rhymes four to six times and sang it two to four times with the experimenter. All tasks of the experiment were integrated in a game: Children had to help a cuddly toy (Paul, the forgetful rabbit) who played musical phrases, but could not remember if he played it correctly or incorrectly. Then they voted using two different buttons, whether the phrase was correct (unmodified) or incorrect (modified). In the melody recognition experiment, they detected which nursery rhyme was played. They gave their response with buttons placed next two a two-by-two array containing pictures representing the four different songs used in this task (see right part of Figure 2).

#### 2.2.1. Melodic Perception

**Figure 1:**
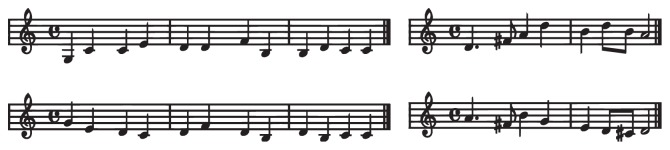
Overview of the stimuli used in the melodic perception (left) and rhythmic-melodic perception (right) tasks. The nursery rhyme used in the melodic perception tasks had the title “Es war eine Mutter, die hatte vier Kinder,” the one for the rhythmic-melodic perception tasks had the title “Alle Vögel sind schon da.” Each task had three conditions: standard (first line), transposed (first line, but played in another key), and comparable (bottom line).

To test melodic perception, the beginning phrase of a well-known nursery rhyme with 12-tone length and a constant rhythm (containing only quarter notes) was used (see Figure 1, left panel). There were three different blocked conditions: standard, transposed, and comparable melody (described below). Within each condition, there were 20 stimuli: In 10 stimuli the melody was not modified; in another 10 stimuli it was changed: in 5 stimuli the tone height was altered while the contour was preserved and in another 5 both tone height and contour were changed. A response was counted correct, if the children detected whether the phrase was unmodified or changed (these two choices were represented by different buttons). Each block lasted about 5 minutes and the whole experiment about 20 minutes. In the standard melody block, the phrase was presented in the original key. In the transposed melody block the melody phrase was presented in five different keys (either original [as in the first block] or one or two half tones up and down). Like in language acquisition, where words and sentences have to be recognized as invariant although they are spoken by different speakers, this task aimed to test the ability to recognize preservation or violation of a melody regardless of whether the key was altered or not. In the comparable melody block, a new melody, previously unknown to the children but similar in harmonic structure and length to the original melody, was introduced in order to test melodic perception while removing the opportunity to subvocally speak the text of the nursery rhyme. The task aimed at testing melodic perception and short-term memory for melodies and was similar to methods used in other tests on musical abilities or musical aptitude (e.g., [43–45]), where children have to compare melodies of which they do not have long-term representations. The novel melody for this task was learned before the experiment by singing it on the syllable [na].

#### 2.2.2. Rhythmic-Melodic Perception

The beginning of another nursery rhyme was used in this task (see Figure 1, right panel). In contrast to the stimuli used in [1], this phrase had a complex rhythmic structure with eighth, quarter, and punctuated quarter notes. In 10 stimuli the original rhythm was kept, while in another 10 stimuli the rhythm was changed at different positions within the phrase (whereas the pitch height was kept constant). This was accomplished, for example, by changing two quarter notes into an eighth and a punctuated quarter note. Comparable to the methodology in the melodic perception part, the stimuli were presented in three block conditions, standard, transposed, and comparable rhythm (similar to those described above). The task took the same amount of time as the first one (20 minutes).

#### 2.2.3. Melody Recognition

**Figure 2:**
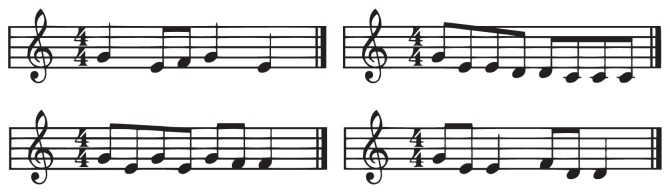
Overview of the stimuli (nursery rhymes) used in the melody recognition task. The stimuli were the nursery rhymes “Hänsel und Gretel” (first row, left), “Der Kuckuck und der Esel” (second row, left), “Weil heute Dein Geburtstag ist” (first row, right), and “Hänschen klein” (second row, right). These stimuli were known to all children.

In the melody recognition task, children had to distinguish the starting phrase of 4 different nursery rhymes (see Figure 2), which were familiar to all children. However, two members of the SLI group did not know all songs and were excluded from the melody recognition task. Each rhyme was represented by a picture arranged in a two-by-two array. Children indicated which song they heard by pressing a button placed next to the picture representing it. Stimuli were modified to create four conditions: They were either played with piano sound in the same key (original); with a piano sound, but in a different key (transposed); with different instrumental timbre (timbre-change); or at a faster tempo (faster). Using these parametric manipulations, we aimed to explore several acoustic features which constitute building blocks of prosodic components. In every condition, each song was played three times, resulting in a total of 12 stimuli in each condition (48 stimuli altogether). To familiarize the children with the task, the experimenter spoke the lyrics of the beginning phrase and children had to press the button placed next to the picture representing the rhyme.

### 2.3. Data Analysis

Using Kolmogorov-Smirnoff tests, we ensured that the analysed variables conformed with a standard normal distribution (SLI: *M* = 0.47; 0.07 ≤ *p* ≤ 0.84; CA: *M* = 0.58; 0.07 ≤ *p* ≤ 0.93; CL: *M* = 0.88; 0.61 ≤ *p* ≤ 1.00). However, given that variance was unequal in the three groups (with a relatively broad range of performance, particularly in children with SLI), we decided to use nonparametric tests to compare the three groups (Mann-Whitney *U* tests) and to explore relations between music perception skills and other variables (Spearman's rank correlations).

First, we compared the results of children with SLI in the language screening and their nonverbal intelligence to those of typically developing children. We also determined, using one-sample *t*-tests, whether the response probabilities were significantly above chance level. Then, we explored whether the experimental groups showed differences in music perception, comparable to the differences in their linguistic abilities. Finally, the performance in music perception tasks was related to that in the language comprehension and working memory subtests of the language screening and variables reflecting their socioeconomic background (given that the status values of their mother's profession could not be perfectly matched).

## 3. Results

Children with SLI performed significantly below their age-equivalent mean scores in all subtests of the language screening (see Table 1; language comprehension: *z* = −1.11; speech production: *z* = −1.05; nonword repetition: *z* = −1.85; sentence repetition: *z* = −1.27; *p* < 0.001). In contrast, the results of the two control groups were slightly above the mean of their population norm (0.18 ≤ *z*_CA_ ≤ 0.63, 0.45 ≤ *z*_CL_ ≤ 0.82). Phonemic discrimination in children with SLI differed from those of comparable age (*p* = 0.006) but not from those with comparable language skills (*p* = 0.154). Although children with SLI had a similar distribution in the range of their IQ scores, nonverbal intelligence scores in children with SLI were below the two control groups (*M*_SLI_ = 92.8; *M*_CA_ = 101.8; *M*_CL_ = 103.1; *p* < 0.01).

**Figure 3:**
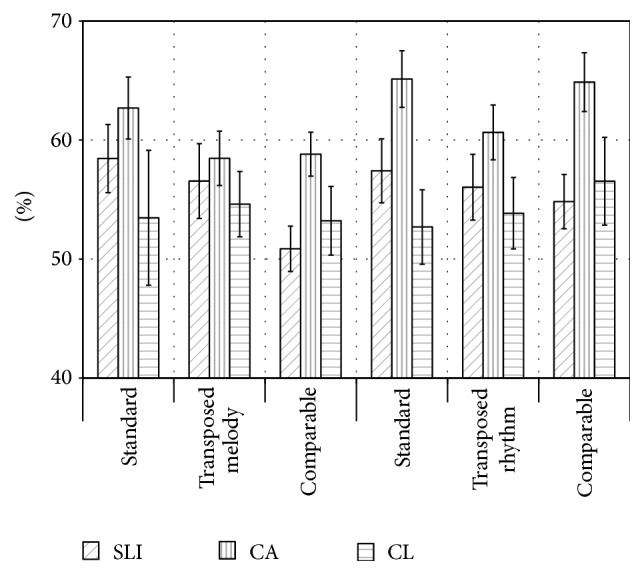
Mean percentage of correct responses (and standard error of mean) for the subtests of the melodic (left panel) and the rhythmic-melodic perception tasks (right panel).

In the melodic perception tasks, children with SLI performed nominally poorer than the age-matched controls (CA) in all conditions (see Figure 3 and Table 2). For the comparable melody condition and the sum score of all three conditions a significant difference between the SLI and the CA group was obtained. The scores of children with SLI were generally in the same range as those of the younger controls (CL). They were nominally slightly higher for the standard and the transposed melody condition. Neither between the children with SLI and the younger controls (CL; *p* > 0.39) nor between the two control groups (*p* > 0.08) significant were differences observed for any condition. The performance of children in the CA group was significantly above chance level in all conditions (*p* ≤ 0.001), the performance of children with SLI did significantly exceed chance in all (0.006 ≤ *p* ≤ 0.046), but the comparable melody condition (*p* = 0.654), and in the CL group performance did not reach above chance levels in any condition (*p* > 0.118; presumably because of the small sample size; as their mean performance was relatively similar to the SLI children).

For the rhythmic-melodic perception tasks (see Figure 3 and Table 2), the CA group showed higher performance levels than the children with SLI in all conditions. These differences were significant for the standard and the comparable rhythm condition as well as for the sum score of all three conditions. Similarly, the CA group showed higher performance levels than the language controls (CL), and significant differences between those two groups were observed for the standard condition and the sum score of all conditions. The performance was significantly above chance for all conditions in children with SLI (0.010 ≤ *p* ≤ 0.043) and the CA group (*p* ≤ 0.001) but not for any condition in the CL group (*p* > 0.101; presumably due to the small sample size).

**Figure 4:**
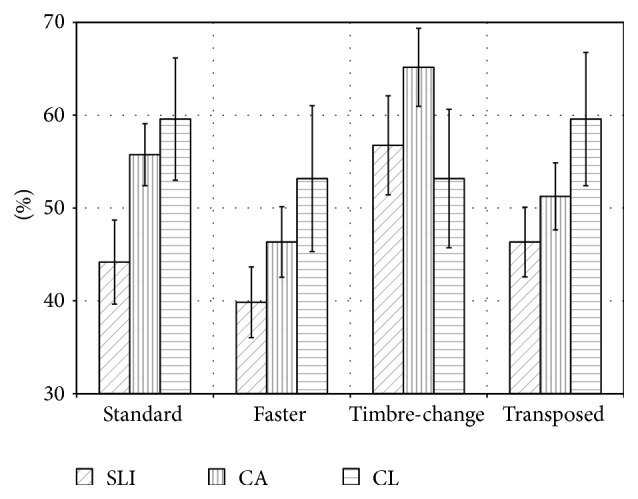
Mean percentage of correct responses (and standard error of mean) for the four subtests of the melody recognition task.

In the melody recognition tasks (see Figure 4 and Table 2), for all conditions except the timbre-change condition, the younger controls (CL) achieved the highest levels of performance, followed by the age-matched controls whereas children with SLI achieved the lowest scores. For the standard condition there was a significant group difference between children with SLI and those of comparable age. For no other condition did a group comparison reach significance. The performance in all groups was above chance level for all conditions (*p* ≤ 0.004).

Using correlation analyses we explored the relationship between the “language comprehension” subtest of the language screening and music perception skills (see Table 3). When the whole group (see left column) is considered, all melody recognition tasks as well as the rhythmic-melodic perception tasks except from the standard condition were significantly correlated with the language comprehension subtest. None of the melody perception tasks were significantly correlated with the language comprehension performance. To ensure that the observed correlations were not simply due to the different skill levels in the three groups, additional correlation analyses were carried out for each group separately. Children with SLI (second column) had significant correlations for the transposed subtest of the rhythmic-melodic perception task, for the sum score of the rhythmic-melodic perception task, and for the faster, and the timbre-change condition as well as the sum score of the melody recognition task. For the control groups (third and fourth column), no correlations between musical and linguistic skills were significant.

Furthermore, we explored correlations among the sum scores of the three music perception tasks and between those sum scores and the results of the psychometric tests and indicators of the socioeconomic status of the family. We observed (see topmost part of Table 4) that the music perception skills were moderately to strongly intercorrelated, indicating that they presumably measure common underlying skills.

Moreover, there are (mainly moderate) correlations between music perception skills and the short-term/working memory subtests of the language screening. No correlations were observed between either the “language comprehension” or the “language production” subtests and melodic perception, whereas those subtests correlated with the rhythmic-melodic perception and the melody recognition tasks. Both music perception tasks also correlated with the hand movements subtest, and rhythmic-melodic perception correlated with the spatial memory subtest of the intelligence test (which assess short-term/working memory). In contrast, no significant correlations were found between music perception skills and measures of the socioeconomic status of the families. That is, regardless of the difference in the status value of the mother's profession, measures of socioeconomic status did not account for the difference in music perception skills in the examined population.

## 4. Discussion

The present study explored music perception in children with SLI and with typical language development in order to determine whether there is a link between speech perception and different aspects of music perception (melodic and rhythmic-melodic perception, as well as melody recognition).

As expected, the groups differed in their linguistic abilities: Children with SLI performed at least 1 SD below their age norm, whereas the control groups performed at the expected age level. For phonemic discrimination, the SLI group had a speech perception level comparable to the CL group, whereas both groups performed below the CA group. Although children with a nonverbal IQ below 80 were excluded from the analysis, the score of children with SLI was still below that of controls.

Notably, children with SLI and those from the control groups differed significantly in their music perception skills. For the previously unknown melodies (comparable condition) and the sum score of all conditions of the melodic perception task, as well as for all subtests except the transposed condition of the rhythmic-melodic perception tasks, the performance level of children with SLI was significantly below that of the age-matched controls and rather similar to that of children with comparable linguistic abilities (CL). Processing of pitch appeared to be easier than that of rhythm: Whereas the differences between the SLI and the CL group were at least approaching significance (*p* ≤ 0.067) for all rhythmic-melodic perception tasks, only for one melodic perception task (comparable) and the sum score a significant group difference was observed.

In the melody recognition tasks, children with SLI performed nominally below both control groups in all but the timbre-change subtest of the melody perception task. However, a significant difference between children with SLI and the CA group was obtained only for the standard condition. While in the melodic and rhythmic-melodic perception tasks (reported above) the performance of children with SLI was similar to the CL group, it is lower (at least nominally) than in either control group for most melody recognition tasks.

Compared to previous evidence, the observation that children with SLI are poorer in detecting violations in melody (pitch) or rhythm (duration) than age-matched controls is similar to results from previous studies [33, 35, 38]. Our data provide a slightly different focus compared to previous studies: Corriveau et al. [33] and Cumming et al. [35] had a stronger focus on rhythm perception and the processing of basic auditory properties (such as amplitude rise time and duration). Clément et al. [38] were primarily interested in the music production skills although they also provided data on music perception skills. Taken together, the data from those studies indicate that the difficulties to process certain acoustic features underlying prosody (amplitude rise time, tempo, stress, and duration) also influence music perception. Importantly, compared to previous studies our data explore a much younger age cohort (four- and five-year-olds as compared to ten- and eleven-year-olds), complementing the knowledge about relations between music perception and linguistic skills at earlier stages of language acquisition.

Significant correlations between the language comprehension subtest and music perception where found when all subgroups are pooled in all conditions of the melody recognition task and for transposed and comparable condition as well for the sum score of the rhythmic-melodic perception tasks but not for the standard condition. Likewise, the sum scores of the music perception tasks were correlated with almost all subtests of the language screening. When exploring the correlations within the group of children with SLI, significant correlations were primarily observed for the more complex conditions where different musical parameters were varied (i.e., the transposed conditions in the rhythmic-melodic perception task, as well as the faster and the timbre-change condition in the melody recognition task). Given that similar correlations can not be observed within either control group and assuming common underlying processing skills during music perception and for prosodic cues (cf., e.g., [22, 23]), these results provide further evidence for the prosodic phrasing hypothesis which suggests that auditory impairments contribute via perceptual difficulties with global prosodic structure to the grammatical difficulties observed in children with SLI. It appears that those skills are more predictive of linguistic proficiency during early language development and in children who are delayed because of impaired language development.

In addition, the correlations of the rhythmic-melodic perception and the melody recognition tasks with those subtests of the intelligence test which assess short-term/working memory suggest that a common underlying general cognitive skill, such as working memory capacity, may account for the correlations between the different music and language perception tasks. However, the difference can not be accounted for by differences in memory load among the stimuli as those had similar acoustic characteristics and length. Baddeley [46] proposed that the capacity of working memory can be increased by automatizing underlying subprocesses. Lack of automaticity in processing musical elements may limit the storage capacity of working memory and hence impede binding the elements of musical phrases together. Assuming that children with SLI have not yet established automatic, preattentive analyses for certain music-specific parameters (melody, rhythm, timbre, etc.) might account for their poor performance, especially when processing complex musical sequences. This assumption has to be further tested in future studies.

One limitation of the current study is that the performance of most children was relatively close to chance level for the melodic and the rhythmic-melodic perception tasks. Particularly the younger controls did not exceed chance level for most tasks, presumably because of the substantial variance in their performance and the rather small sample size. The development of tasks that can be accomplished by younger children would thus be desirable.

Our results suggest that exploring music perception skills can inform theories about typical and impaired language acquisition. For this reason, the tasks used in the present study should be further developed: Musical material can be a useful indicator of language processing difficulties, because it allows exploring skills that are prerequisites of successful language perception. Parameters or features like pitch, timbre, tempo, and their complexity can be easily manipulated using musical material. A fine-grained assessment of musical skills and a detailed description of their relation to linguistic abilities can provide important information about aetiology of SLI and may open new perspectives for diagnosis and therapy of those children.

## 5. Conclusion

The current study demonstrated difficulties of children with SLI to perceive changes in the pitch and rhythm of musical phrases. It complements and extends evidence from previous studies that observed difficulties with processing musical syntax [36], perceiving rhythm [33, 35], and producing music [37, 38]. This suggests that children with SLI also show difficulties with aspects of nonlinguistic processing which are potentially a crucial phenomenon of SLI. The observed relations between language and music perception strengthen assumptions about the importance of musical parameters during language acquisition [9, 10].

## Figures and Tables

**Table 1:** Summary of characteristics of the three groups of participants (SLI: Children with Specific Language Impairment; CA: children of comparable age; CL: children with comparable linguistic abilities) encompassing age and gender distribution, performance in psychometric tests (language screening and general cognitive skills), and variables reflecting the socioeconomic background. (*T*): *T*-scores, (*S*): standard scores, (*R*): raw scores.

	SLI (*N* = 29)	CA (*N* = 39)	CL (*N* = 13)
Age (in months)	64.2 (56–71)	63.6 (57–71)	51.3 (48–55)
Gender (male/female)	19/10	22/17	8/5
Psychometric tests			
Language comprehension (*T*)	38.9 (20–59)	51.8 (39–72)	54.5 (40–74)
Language production (*T*)	39.5 (21–79)	56.3 (40–79)	58.2 (45–72)
Nonword repetition (*T*)	31.5 (20–61)	53.0 (35–68)	56.4 (35–70)
Sentence repetition (*T*)	37.3 (20–58)	55.0 (41–74)	55.3 (39–63)
Phoneme discrimination (*R*)	9.8 (3–17)	12.6 (7–17)	11.8 (6–16)
Nonverbal IQ (*S*)	91.8 (81–115)	101.8 (84–120)	103.1 (85–125)
Socioeconomic status			
Mother's occupation (ISEI)	34.2 (16–52)	42.8 (25–66)	50.4 (29–73)
Father's occupation (ISEI)	34.5 (23–71)	37.4 (19–69)	39.4 (29–73)
Children's books	45.4 (1–207)	46.4 (4–138)	42.5 (1–102)
Children's CDs	25.2 (0–88)	21.6 (5–55)	23.3 (7–70)

**Table 2:** The first three columns contain mean percentages of correct responses for children with Specific Langugage Impairment (SLI), children of comparable age (CA), and children with comparable linguistic abilities (CL). In the last three columns significance levels of the group comparisons (using the Mann-Whitney *U* tests) are given. Results for melodic perception are in the top section, rhythmic-melodic perception in the middle, and melody recognition at the bottom. Significant group differences are written bold.

	Correct responses			Group comparison		
	SLI	CA	CL	SLI-CA	SLI-CL	CA-CL
Melodic perception						
Standard	58.45%	62.70%	53.45%	0.306	0.519	0.161
Transposed	56.55%	58.45%	54.60%	0.578	0.648	0.259
Comparable	50.86%	58.81%	53.21%	**0.003**	0.389	0.156
Sum score	53.85%	59.23%	52.82%	**0.040**	0.979	0.081
Rhythmic-melodic perception						
Standard	57.40%	65.15%	52.70%	**0.014**	0.451	**0.006**
Transposed	56.05%	60.65%	53.85%	0.067	0.872	0.135
Comparable	54.85%	64.85%	56.55%	**0.004**	0.519	0.106
Sum score	56.10%	63.55%	54.37%	**0.003**	0.591	**0.010**
Melody recognition						
Standard	44.17%	55.75%	59.58%	**0.033**	0.073	0.678
Faster	39.83%	46.33%	53.17%	0.356	0.187	0.558
Timbre-change	56.75%	65.17%	53.17%	0.205	0.732	0.148
Transposed	46.33%	51.25%	59.58%	0.420	0.089	0.293
Sum score	46.75%	54.65%	56.42%	0.110	0.177	0.866
Spoken (control)	93.83%	98.08%	96.17%	0.113	0.955	0.130

**Table 3:** Correlations of all music perception subtests with the language comprehension subtest of the language screening (significance level in parentheses) for all groups of children (first column) as well as each subgroup separately (second to fourth column). Significant correlations are indicated by bold typeface.

	All children (*N* = 81/79)		SLI (*N* = 29/27)		CA (*N* = 39)		CL (*N* = 13)	
Melodic perception								
Standard	0.10	(0.357)	0.06	(0.764)	0.03	(0.840)	0.17	(0.579)
Transposed	0.13	(0.242)	0.26	(0.169)	0.07	(0.684)	−0.03	(0.931)
Comparable	0.19	(0.097)	0.07	(0.715)	0.10	(0.559)	−0.14	(0.655)
Sum score	0.20	(0.075)	0.14	(0.474)	0.08	(0.628)	0.00	(0.993)
Rhythmic-melodic perception								
Standard	0.16	(0.166)	0.11	(0.578)	0.02	(0.908)	−0.06	(0.845)
Transposed	**0.26**	**(0.017)**	**0.47**	**(0.010)**	−0.03	(0.855)	0.23	(0.448)
Comparable	**0.32**	**(0.004)**	0.18	(0.354)	0.22	(0.189)	−0.06	(0.853)
Sum score	**0.31**	**(0.005)**	**0.38**	**(0.044)**	0.11	(0.511)	0.01	(0.964)
Melody recognition								
Standard	**0.28**	**(0.014)**	0.33	(0.091)	0.08	(0.628)	0.36	(0.227)
Faster	**0.29**	**(0.011)**	**0.39**	**(0.043)**	0.26	(0.113)	0.26	(0.383)
Timbre-change	**0.40**	**(<0.001)**	**0.52**	**(0.005)**	0.25	(0.118)	0.53	(0.065)
Transposed	**0.25**	**(0.029)**	0.28	(0.153)	0.22	(0.171)	0.35	(0.239)
Sum score	**0.33**	**(0.003)**	**0.45**	**(0.019)**	0.19	(0.236)	0.39	(0.188)

**Table 4:** Intercorrelations of the music perception subtests (top part), as well as correlations of the music perception subtests with the subtests of the language screening (middle part) and with indicators of the socioeconomic background of the participants. Correlations are reported for the whole group (SLI, CA, and CL), their significance level is shown in parentheses, and significant correlations are indicated by bold typeface.

	Melodic perception		Rhythmic-melodic perception		Melody recognition	
Melodic perception			**0.64**	**(<0.001)**	**0.36**	**(0.001)**
Rhythmic perception					**0.45**	**(<0.001)**
Language comprehension	0.20	(0.075)	**0.31**	**(0.005)**	**0.33**	**(0.003)**
Language production	0.11	(0.313)	**0.24**	**(0.033)**	**0.29**	**(0.009)**
Nonword repetition	**0.32**	**(0.004)**	**0.42**	**(<0.001)**	**0.37**	**(0.001)**
Sentence repetition	**0.31**	**(0.002)**	**0.49**	**(<0.001)**	**0.41**	**(<0.001)**
Hand movements	0.17	(0.139)	**0.32**	**(0.004)**	**0.24**	**(0.034)**
Spatial memory	0.20	(0.068)	**0.24**	**(0.030)**	−0.01	(0.944)
Mother's ISEI score	0.05	(0.692)	0.00	(0.972)	0.14	(0.261)
Father's ISEI score	−0.12	(0.374)	−0.04	(0.740)	0.04	(0.750)
Books in household	0.04	(0.731)	0.04	(0.735)	0.12	(0.315)
CDs in household	0.18	(0.121)	0.16	(0.183)	0.16	(0.190)
